# Mitochondrial Dysfunction, Oxidative Stress, and Neuroinflammation: Intertwined Roads to Neurodegeneration

**DOI:** 10.3390/antiox9080647

**Published:** 2020-07-22

**Authors:** Anna Picca, Riccardo Calvani, Hélio José Coelho-Junior, Francesco Landi, Roberto Bernabei, Emanuele Marzetti

**Affiliations:** 1 Fondazione Policlinico Universitario “Agostino Gemelli” IRCCS, 00168 Rome, Italy; anna.picca@guest.policlinicogemelli.it (A.P.); francesco.landi@unicatt.it (F.L.); emanuele.marzetti@policlinicogemelli.it (E.M.); 2 Università Cattolica del Sacro Cuore, 00168 Rome, Italy; coelhojunior@hotmail.com.br

**Keywords:** Alzheimer’s disease, cytokines, DAMPs, Down syndrome, endo-lysosomal system, extracellular vesicles, mitochondrial DNA, mitochondrial quality control, mitophagy, Parkinson’s disease

## Abstract

Oxidative stress develops as a response to injury and reflects a breach in the cell’s antioxidant capacity. Therefore, the fine-tuning of reactive oxygen species (ROS) generation is crucial for preserving cell’s homeostasis. Mitochondria are a major source and an immediate target of ROS. Under different stimuli, including oxidative stress and impaired quality control, mitochondrial constituents (e.g., mitochondrial DNA, mtDNA) are displaced toward intra- or extracellular compartments. However, the mechanisms responsible for mtDNA unloading remain largely unclear. While shuttling freely within the cell, mtDNA can be delivered into the extracellular compartment via either extrusion of entire nucleoids or the generation and release of extracellular vesicles. Once discarded, mtDNA may act as a damage-associated molecular pattern (DAMP) and trigger an innate immune inflammatory response by binding to danger-signal receptors. Neuroinflammation is associated with a large array of neurological disorders for which mitochondrial DAMPs could represent a common thread supporting disease progression. The exploration of non-canonical pathways involved in mitochondrial quality control and neurodegeneration may unveil novel targets for the development of therapeutic agents. Here, we discuss these processes in the setting of two common neurodegenerative diseases (Alzheimer’s and Parkinson’s disease) and Down syndrome, the most frequent progeroid syndrome.

## 1. Introduction

Oxidative stress and neuroinflammation are pathologic signatures of neurodegeneration [1,2]. Cells of innate immunity are a source of reactive oxygen species (ROS) within the central nervous system (CNS), with oxidant overproduction being a candidate mechanism in neuronal damage and loss via neuroinflammation [3,4]. The molecular determinants of immune-mediated oxidative stress and neurodegeneration remain poorly understood. However, microglia activation is considered to be a major contributor [3,5]. 

Microglial cells are CNS macrophages that ensure homeostasis of the nervous tissue by clearing out damaged neurons and limiting the spread of infections. As a first line of defense against microbes, this macrophage population triggers inflammation via cytokine production and instigates ROS generation [6]. However, a long-standing pro-inflammatory and pro-oxidant environment sustained by persistent microglia activation is detrimental as it may contribute to neurodegeneration. This is particularly relevant in the context of Alzheimer’s (AD) and Parkinson’s disease (PD) [7,8]. Metabolic changes in microglia have also been associated with a state of chronic low-grade inflammation observed during aging (i.e., inflamm-aging) [9,10]. Oxidative stress is also a hallmark of progeroid conditions. For instance, Down syndrome (DS), caused by a genetic imbalance of chromosome 21 which contains genes coding for enzymes implicated in oxidative stress (e.g., Cu/Zn superoxide dismutase, SOD1), is characterized by a breach in antioxidant defenses [11,12]. What is more relevant, people with DS, among other clinical features, show cognitive deficits and are prone to developing AD [13]. Hence, a deeper understanding of the molecular determinants of progeroid conditions may provide relevant insights into the pathophysiology of age-related neurodegenerative conditions as they may share common roots.

Mitochondria are a major source of ROS as a byproduct of the electron transport chain (ETC) activity [14]. These organelles are also a direct ROS target [14]. Oxidative stress-related mitochondrial disarrangements have been indicated as contributors to neurodegeneration [15]. Only recently, it has emerged that, apart from their relevance to cellular energy provision and ROS signaling, mitochondria participate in inflammation by triggering a danger signaling response [16,17]. In particular, in response to various stressors, mitochondrial DNA (mtDNA) can be displaced into intra- or extracellular compartments [18]. Albeit the mechanisms whereby mtDNA is unloaded from mitochondria are largely unclear, several lines of evidence indicate that mtDNA can be shuttled toward the extracellular compartment via the generation and release of extracellular vesicles (EVs) [18,19,20,21]. Along with mtDNA and other mitochondrial components, cell-free intact mitochondria have been found in the blood of healthy people [21]. What is more, these organelles show functional competence [21]. Once released, mitochondrial constituents, in particular mtDNA, may act as damage-associated molecular patterns (DAMPs) that can be sensed as non-self molecules and trigger an innate immune inflammatory response by binding to danger signal receptors [16,17].

Further to this, mitochondria take part in a highly interconnected and dynamic network of organelles and structures, including the endoplasmic reticulum, lysosomes, and the actin cytoskeleton [22,23]. Mitochondrial plasticity is also influenced by energy-sensing mediators including insulin, insulin-like growth factor 1 (IGF1), mechanistic target of rapamycin (mTOR), AMP-activated protein kinase (AMPK), and Sirtuins [24]. Metabolic alterations arising from deregulated nutrient sensing have been reported in the setting of neurodegeneration in conjunction with mitochondrial dysfunction and oxidative stress [24]. Indeed, mitochondrial homeostasis relies upon the integrity of intracellular organelle networks, inter-mitochondrial content exchange, and integration of metabolic signaling [22,24]. Via inter-mitochondrial junctions, adjacent organelles coordinate membrane cristae remodeling [25], while mitochondrial fusion allows matrix and intermembrane space content (i.e., proteins, mtDNA, and metabolites) to be mixed between organelles to avoid focal accumulation of damaged constituents [26]. Recently, tubular protrusions, referred to as mitochondrial nanotunnels, have been identified as novel structures enabling mitochondrial interconnections [27]. Via these structures, mitochondria that are immobilized within post-mitotic tissues (e.g., skeletal muscle, myocardium) and limited in their fusion events, may communicate over long distances [27]. Finally, Golgi-derived vesicles have also been shown to participate in mitochondrial dynamics and homeostasis [28].

Neuroinflammation is associated with a large set of neurological disorders for which mitochondrial derived vesicles (MDVs) may represent a common thread supporting disease progression. The analysis of these pathways may help clarify the events linking cell dyshomeostasis with peripheral changes that could even precede neuroinflammation. Indeed, EVs are shuttle systems whereby cells interact or remove unwanted materials [29]. In the setting of failing mitochondrial fidelity pathways, MDVs serve a further layer of mitochondrial quality control (MQC) orchestrated by mitochondrial–lysosomal crosstalk [20]. The clearance of dysfunctional organelles via MDVs involves the release of noxious material that can potentially trigger sterile inflammation. This response is part of innate immunity and operates through the binding and activation of membrane or cytoplasmic pattern recognition receptors (PRRs) (reviewed in [30]). Indeed, mtDNA possesses hypomethylated CpG motifs that are similar to those of bacterial DNA and are recognized by PRRs [30]. 

In this context, a comprehensive profiling of neuronal-derived DAMPs, including those transported within MDVs, offers the opportunity to shed light on the complex regulatory network governing intracellular homeostasis. This knowledge may also allow deciphering peripheral changes that accompany neurodegeneration. The relevance of EVs in shuttling DAMPs resides in the unique packaging of the information contained, which confers protection of the cargo and delivery of multiple messengers in a paracrine and endocrine fashion. The exploration of these non-canonical pathways arising from mitochondrial dysfunction and contributing to neurodegeneration may unveil novel targets for the development of therapeutics. Here, we discuss these pathways in the setting of two common neurodegenerative diseases (AD and PD) and DS, the most frequent progeroid syndrome.

## 2. Mitochondrial Quality Control, mtDNA Release, and Inflammation

### 2.1. Failing Mitochondrial Quality Control and mtDNA Release

A set of MQC processes spanning from mitochondriogenesis to mitochondrial plasticity, autophagy, and mitochondrial unfolded protein response (UPR^mt^) are in place to safeguard cell and organismal homeostasis [20]. Mitochondrial dynamics rely upon coordinated cycles of fusion and fission. An adequate mitochondrial plasticity is crucial for matching cellular energy demands and diluting mitochondrial damage along the network [26]. Changes in mitochondrial dynamics are sensed and integrated by a nutrient-sensing signaling pathway that modulates mitochondrial functions via a set of energy-sensing mediators (e.g., insulin, IGF1, mTOR, AMPK, Sirtuins) [24]. When mitochondria become bioenergetically incompetent, mitochondrial hyper-fission segregates damaged organelles from the network and primes them for subsequent disposal via mitophagy [26]. Concomitantly, mitochondrial biogenesis, through generating new organelles, maintains an adequate pool of functional mitochondria within the cell [26]. Mitochondrial epigenetic regulation (i.e., mito-epigenetics) is also relevant for proper cell signaling, as shown by the existence of specific patterns of mtDNA methylation associated with susceptibility to developing neurodegenerative diseases [31]. Mito-epigenetic control over genes encoding mitophagy factors has also been implicated in longevity via modulation of UPR^mt^ signaling [32,33]. 

Failing MQC is a major contributor to aging and associated conditions, including neurodegeneration [24]. In this context, circulating mtDNA is increasingly acknowledged as a pivotal mediator linking mitochondrial dysfunction to inflammation [30,34,35]. Of all mitochondrial components, mtDNA is the most susceptible to be sensed as a “non-self” molecule due to the presence within its structure of aberrant/devoid CpG methylation motifs that can trigger inflammation [34,35,36,37,38,39,40]. Fragmented mtDNA as well as circular double-stranded or linear single-stranded mtDNA molecules and nucleoids have been found in the human plasma in various disease conditions [37,38,41,42,43]. However, the mechanisms regulating the displacement of mtDNA into the extracellular compartment are still fuzzy. Among the most accredited hypotheses, an unconventional unloading of damaged mtDNA during stressful conditions seems to occur as part of the danger signaling response to support cell’s homeostasis [16,17]. 

**Figure 1:**
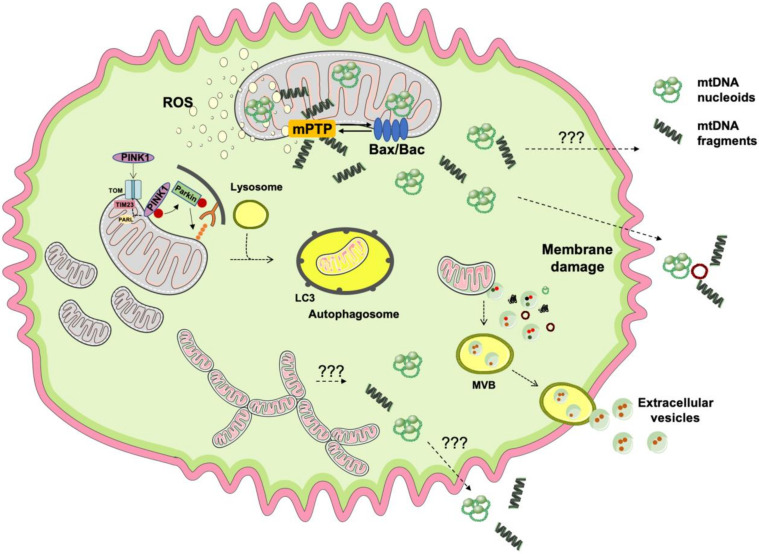
Schematic representation of the pathways involved in mitochondrial DNA unloading. Under several stressors (e.g., mitochondrial dysfunction and consequent reactive oxygen species (ROS) production, viral and bacterial infections), several types of damage are inflicted to mitochondrial DNA (mtDNA) and a set of signaling pathways are activated to promote mitochondrial recovery or demise depending on the damage severity. (1) In the setting of ROS bursts, mtDNA oxidation and fragmentation occur concomitant with mitochondrial permeability transition pore (mPTP) opening. If the extent of damage overwhelms the mitochondrial quality control system, persistent mPTP opening allows extrusion of oxidized mtDNA fragments into the cytosol. Meanwhile, the deployment of apoptosis via Bax/Bak signaling enables the extrusion of mtDNA nucleoids consisting of mitochondrial transcription factor A-bound mtDNA. (2) Transient opening of mPTP and ROS production trigger mitochondrial fission and mitophagy to clear dysfunctional mitochondria. Damaged organelles are sequestered into autophagosomes that subsequently fuse with lysosomes for content disposal. Herein, a set of DNases execute mtDNA cleavage. (3) Hyperfragmented or hyperfused mitochondria also deliver mtDNA into the cytosol from which it can be extruded into the extracellular space through unknown mechanisms. (4) If mitochondria are only mildly damaged, mtDNA and other mitochondrial constituents can be shuttled outside the cell via extracellular vesicles (EVs). EVs of mitochondrial origin (i.e., mitochondrial derived vesicles) may fuse with multivesicular bodies (MVBs) and deliver their cargo in the extracellular space for signaling purposes. (5) Following cell injury or necrosis, the damaged plasma membrane may represent a route for the delivery of mtDNA and other cellular components outside of the cell. *Abbreviations*: LC3, microtubule-associated protein 1A/1B-light chain 3; PARL, presenilin-associated rhomboid-like; PINK, phosphatase and tensin homolog-induced kinase 1; TIM, translocase of the inner mitochondrial membrane; TOM, translocase of the outer mitochondrial membrane.

Along with other insults, the installment of oxidative stress as a result of ROS overproduction mainly by mitochondria is a known trigger of inflammation [44]. The activation of redox-sensitive inflammatory pathways has been reported in case of altered mitochondrial calcium metabolism, iron mishandling, and higher ROS production [45,46]. ROS bursts are a major pro-inflammatory stimulus *per se* via activation of nuclear factor κB (NF-κB) [47]. However, mitochondrial ROS also participate in other cellular processes including the activation of mitochondrial permeability transition pore (mPTP), dysregulation of mitophagy, imbalances in mitochondrial dynamics, and programmed cell death [48]. These processes represent potential routes for mtDNA displacement following ROS burst as evidenced by the finding of oxidized mtDNA fragments outside of mitochondria in the setting of several metabolic diseases [43,49,50,51,52,53] (Figure 1).

Under oxidative stress conditions, mPTP opening induces a transient increase of inner mitochondrial membrane permeability which allows the release of ions, small molecules, and mitochondrial matrix components, including mtDNA [54]. Instead, irreversibly damaged and uncoupled mitochondria undergo permanent mPTP opening which also has been implicated in mtDNA extrusion [55]. In both cases, the release of mtDNA via mPTP from isolated mitochondria is blunted by the administration of ciclosporin A (CSA) [42,56]. In vitro experiments have clarified that, under oxidative stress, the unloading of mtDNA fragments is attributable to permanent mPTP activation [42,56]. Indeed, in isolated mitochondria exposed to oxidative stress, the release of mtDNA but not its fragmentation is attenuated by CSA [42,56].

Along with mPTP opening, oxidative stress can also activate mitophagy for organelle disposal [57,58]. Mitophagy involves the engulfment of dysfunctional mitochondria into autophagosomes, which then fuse with lysosomes for organelle degradation [59]. Derangements of the mitophagic machinery may result in the accrual of damaged mitochondrial constituents that can follow alternative clearance routes. Mitochondrial components can be released into the cytosol or in the extracellular space where they trigger an innate immune response [39,52,60,61,62]. Of note, any insults overwhelming the mitophagic process can induce inflammation [60,61]. Hence, the cell’s ultimate fate depends on the severity of the inflammatory response and the efficiency of cellular quality control systems. While moderate inflammatory stimuli and defective cellular repair may trigger apoptosis, in the context of persistent inflammation, mitochondrial dysfunction and ROS-induced necrosis may ensue [63]. Indeed, mildly damaged mitochondria can trigger a transient mPTP opening [57,58]. Instead, apoptosis is executed when mitochondrial damage is too severe and/or mitophagy is overwhelmed [57,64]. In the case of programmed cell death, the activation of the Bax/Bak pathway may induce permeabilization of the outer mitochondrial membrane with the formation of pores that, under prolonged stress, become deeper until reaching the inner mitochondrial membrane. Via these structures, mtDNA nucleoids (i.e., mtDNA complexed with the mitochondrial transcription factor A) can be displaced into the cytosol [37,38,65]. Altered mitochondrial dynamics have also been associated with mtDNA-driven inflammation [34,35,36,39,40]. In particular, preclinical models of optic atrophy 1 ablation and dynamin related protein 1 (Drp1) overexpression show hyper-fragmented mitochondria and giant mtDNA nucleoids outside of mitochondria that are able to trigger an immune response [66].

Emerging evidence indicates that the release of EVs from mitochondria (MDVs) occurs independent of mitochondrial depolarization, autophagy, or mitochondrial fission [67]. Indeed, MDVs are generated also in cells lacking autophagy-related serine/threonine kinase gene 5, Beclin-1 or the endolysosomal regulator Ras-related in Brain protein 9 (RAB9) as well as in cells silenced for DRP1 [67]. However, MDV biogenesis requires priming by the mitophagy factors, phosphatase and tensin homolog-induced kinase 1 (PINK1) and Parkin [68]. Therefore, the release of MDVs may represent a complement to mitophagy for MQC when canonical degradative pathways are overwhelmed or impaired [69]. A proposed mechanism for MDV formation involves the accrual of damaged mitochondrial components in proximity of mitochondrial membranes under oxidative stress conditions [68]. This event, along with cardiolipin oxidation, may inflict curvatures-like alterations to mitochondrial membranes that are thought to compete with the activity of import channels [68]. Following the formation of membrane curvatures, the accumulation of PINK1 at the outer mitochondrial membrane and ubiquitination and recruitment of Parkin occur [68]. A set of unidentified proteins would finalize the process, eventually culminating in the release of a vesicle [68]. Hence, all cell constituents, including those of mitochondrial origin, may potentially be extruded into the circulation as DAMPs in the form of cell-free molecules or packaged within vesicles [19].

### 2.2. mtDNA Release and Inflammation

**Figure 2:**
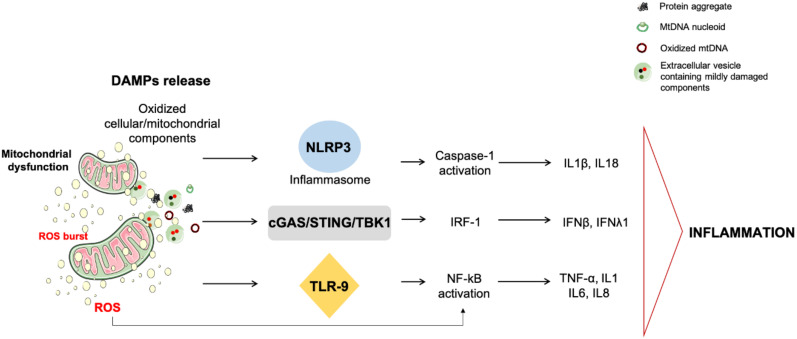
Signaling pathways whereby damaged-associated molecular patterns can trigger inflammation. Failing mitochondrial quality control processes may lead to intracellular accrual of damaged components and their release as damaged-associated molecular patterns (DAMPs). Acting as a DAMP, oxidized mitochondrial DNA (mtDNA) either in the form of mitochondrial transcription factor A-bound nucleoids (green circles) or free mtDNA (red circles) can trigger inflammation via interacting with (1) toll-like receptors (TLRs), (2) nucleotide-binding oligomerization domain (NOD)-like receptor family pyrin domain containing 3 (NLRP3) inflammasome, and (3) cytosolic cyclic GMP/AMP synthase (cGAS)–stimulator of interferon genes (STING) DNA-sensing system. *Abbreviations*: IFN, interferon; IL, interleukin; IRF-1, interferon regulatory factor 1; mtDNA, mitochondrial DNA; NF-κB, nuclear factor κB; ROS, reactive oxygen species; TBK1, TRAF family member-associated NF-κB activator (TANK)-binding kinase 1; TNF-α, tumor necrosis factor alpha.

Among mitochondrial DAMPs that can be released by the cell, mtDNA is a potent trigger of the innate immunity response due to its bacterial ancestry and the presence of hypomethylated CpG motifs [70,71]. Indeed, mtDNA instigates inflammation via the interaction with PRRs, including toll-like receptors (TLRs), nucleotide-binding oligomerization domain (NOD)-like receptors (NLRPs), and the cyclic GMP/AMP synthase–stimulator of interferon genes (cGAS–STING) systems [72,73] (Figure 2).

Mitochondrial DNA can engage the TLR pathway by binding TLR9 at the endo-lysosomal level. As a consequence of this interaction, TLR9 recruits the innate immune signal transduction adaptor myeloid differentiation primary response 88 (MyD88) that activates the mitogen-activated protein kinase and instigates inflammation via NF-κB signaling [74,75,76]. Through the same pathway, neutrophil recruitment and NF-κB-triggered inflammation occur [74,75,76]. Alternatively, mtDNA can induce inflammation via inflammasome and cGAS–STING activation within the cytosol [77,78,79,80,81].

NLRP3 is the best characterized multi-subunit inflammasome system. Upon activation via binding to adaptor molecules, NLRP3 engages caspase-1 and promotes caspase-1-dependent cleavage and activation of interleukin (IL) 1 and 18 [82]. NLRP3-induced inflammation has been observed in several conditions, including AD (reviewed in [83]). The synergistic activation of redox-sensitive inflammation and inflammasome concurs to reinforcing the inflammatory response [84]. The molecular determinants linking inflammasome activation to sterile inflammation are unclear; though, the presence of bacterial-like motifs in mtDNA are sensed by NLRs [85]. NLRP3 activation has also been indicated to act as an upstream checkpoint of the innate immune system during the deployment of sterile inflammation. Indeed, NLRP3 facilitates mPTP opening, thereby contributing to mtDNA release [62]. Hence, a self-sustaining circle of mitochondrial dysfunction, ROS bursts, and mtDNA damage triggered by NLRP3 activators has been hypothesized [78]. Oxidized mtDNA, once released, is preferentially bound and may serve as the ultimate NLRP3 ligand [78], thus confirming the crucial role played by ROS in inflammasome activation [77].

The cGAS–STING DNA-sensing pathway also contributes to sterile inflammation as part of an innate immunity response driven by mtDNA [73]. In particular, the binding of mtDNA to cGAS induces a conformational change that enables the recruitment of STING protein at the endoplasmic reticulum and the subsequent phosphorylation of the transcription factor, interferon (IFN) regulatory factor 3 (IRF-3) via TRAF family member-associated NF-κB activator (TANK)-binding kinase (TBK). Once activated, IRF-3 triggers the expression of type I and III IFN and IFN-stimulated nuclear gene. The activation of cGAS–STING DNA pathway by mtDNA has been observed in the setting of cell death, mitochondrial stress, and viral infections [79,80,81]. 

The cGAS–STING-DNA driven inflammation has been implicated in neurodegeneration following mitophagy impairment [52]. Higher circulating levels of proinflammatory IL6 and INFβ were found in Pink and Parkin knockout mice when challenged with exhaustive exercise compared with wild-type littermates [52]. Notably, this response is blunted by deleting STING or by administering INFα/β receptor-blocking antibody, thus suggesting that the accrual of dysfunctional mitochondria may be necessary for the release of mtDNA to drive inflammation in PD [52].

Following the view of altered quality control as a source of DAMPs, in the next paragraphs, we discuss the main literature on mitophagy impairment and mtDNA dyshomeostasis in the setting of two common neurodegenerative diseases (AD and PD) and DS, the most frequent progeroid syndrome.

## 3. Alzheimer’s Disease

AD is the most common age-related dementia and is featured by neuronal loss mainly in the neocortex and the hippocampus [28]. The deposition of extracellular plaques consisting of amyloid beta (A*β*) aggregates and intracellular neurofibrillary tangles are major histopathological features of the disease [28]. The deposition of these noxious proteins in the brain is associated with and contribute to cognitive impairment [86]. A*β* aggregates are produced via proteolytic cleavage of the C-terminus of the amyloid precursor protein (APP) by *β*- and *γ*-secretase, while neurofibrillary tangles mainly consist of hyperphosphorylated and misfolded Tau protein [86]. Although AD cases are mostly sporadic, mutations in APP or presenilin, the catalytic subunit of *γ*-secretase, have been found in familial forms of AD [87]. 

Following amyloid plaque deposition, the activation of microglia and the release of inflammatory cytokines, including IL1*β*, IL6 and tumor necrosis factor alpha (TNF-α), have been documented in AD [88]. A role for these inflammatory markers in inter- and intracellular signaling in microglia and astrocytes has been hypothesized in AD [89]. Indeed, in the absence of efficient intracellular quality control [90], the persistence of damaged components and local inflammation may induce neurotoxicity and favor the generation of A*β* peptides [88]. 

Alterations of the endo-lysosomal system contribute to the pathogenesis of AD. Aggregates of A*β* peptides, in particular the most pathogenic A*β*42 aggregate, have been detected in the soma of neurons at the level of lysosomes or lysosome-derived components [91]. Moreover, enlarged and dysfunctional multivesicular bodies (MVBs), a specialized type of late degradative endosome, have been described in neurons from AD transgenic mice in the presence of bulky levels of A*β*42 [92]. Such an impairment was found to affect the whole endo-lysosomal system as A*β* accrual was also detected in the enlarged endocytic compartment [92]. Moreover, following MVB dysfunction, an increased APP secretion occurs in the extracellular compartment of these transgenic mice [92]. The endosomal compartment is also a hub for the intracellular trafficking of APP by hosting the activity of the β-site APP-cleaving-enzyme, which is involved in amyloid plaque generation [93]. Indeed, a retromer complex is operative to allow retrograde transport of APP from endosomes to the trans Golgi network and reduces A*β* production [94]. Instead, impaired retromer complex activity has been involved in AD pathogenesis [94]. 

Mitochondrial dysfunction and associated oxidative stress have been implicated in AD pathophysiology. More specifically, decreases in neuronal mtDNA copy number and higher mtDNA heteroplasmy have been described in post-mortem brains of people with AD [95,96]. Off-target reads from high-depth whole exome mtDNA sequencing further confirmed the presence of lower mtDNA content in the brain of persons with AD [97]. Moreover, a focal accumulation of iron was detected in several brain areas at sites of AD lesions, lending further support to the hypothesis of redox-generated free radicals as relevant contributor to AD [98]. Age-associated iron dyshomeostasis has also been associated to loss of mtDNA stability in human skeletal muscle following oxidative stress [46]. More relevant is the observation that oxidative damage to mitochondrial proteins and DNA is an early event in AD, thus suggesting a role for oxidative stress in disease progression [99,100]. A*β* peptide aggregates and neurofibrillary tangles have been implicated in mitochondrial dysfunction as these aberrant proteins can bind to proteins of the mitochondrial import machinery, thereby impairing mitochondrial homeostasis [101]. The interaction of these proteins with mitochondria is detrimental to the ETC activity and results in increased ROS production [102]. Mitochondrial dysfunction and oxidative stress have also been associated with mitochondrial localization of fragments of APOE4, the E4 variant of apolipoprotein E, which is the main susceptibility gene for sporadic AD, in hippocampal neurons [103,104].

Although mitochondrial dysfunction may occur as a primary organelle deficit, aberrant mitochondria can also stem from defective quality control mechanisms, especially mitophagy. In this context, neuronal bioenergetic failure may be linked to inflammation and neuronal loss [105]. Indeed, the generation of A*β* plaque and phosphorylated-Tau (p-Tau) may install a vicious circle between defective mitophagy and mitochondrial dysfunction, ultimately leading to neuronal disruption [105,106,107]. This may ensue from impaired mitochondrial proteostasis via disarrangements of the UPR^mt^ machinery and may represent a link between mitochondrial dyshomeostasis and A*β* proteotoxicity [108]. In this context, the activating transcription factor associated with stress 1 (ATFS-1) may play a relevant role [109]. Indeed, ATFS-1 is either translocated into the mitochondrial matrix to inhibit the UPR^mt^ or relocated into the nucleus to trigger mitochondrial turnover depending on organelle health [109]. An altered expression of the mitophagy receptor disrupted-in-schizophrenia 1 (DISC1) has been found in AD patients, transgenic AD mice model, and A*β*-treated cultured cells [110]. DISC1 promotes mitophagy via binding to microtubule-associated proteins 1A/1B light chain 3 and by protecting synaptic plasticity from A*β* accumulation-induced toxicity [110].

Further support to a role for defective mitophagy in AD pathogenesis is lent by the positive effect of pharmacological restoration of mitophagy on cognitive dysfunction and A*β* proteinopathy in APP/PS1 mice [111]. Moreover, a reduced Tau phosphorylation and mitigation of microglia-induced inflammation have been observed following pro-mitophagy pharmacological treatments [106]. Mitophagy derangements and consequent mitochondrial dysfunction in AD may sustain the extrusion of dysfunctional mitochondrial constituents followed by stimulation of innate immunity [72,73]. Mitochondrial DAMPs have been identified as part of circulating EVs in age-related conditions, including neurodegeneration [112,113]. Should this pathway also be operative in AD, it may help explain the installment of systemic inflammation in the context of mitochondria dysfunction in AD [114]. In-depth characterization of EVs cargo may also shed light onto the pathways responsible for translocation of A*β*42, T-Tau, P-Tau, and neurofilament light polypeptide into the circulation in AD [115].

## 4. Parkinson’s Disease

PD is the second most common neurodegenerative disorder affecting older adults [116]. The progressive loss of dopaminergic neurons of the substantia nigra pars compacta and dopamine depletion in the striatum characterize PD [117]. These histopathological and biochemical features manifest as motor (i.e., bradykinesia, postural instability, rigidity, and tremor) and non-motor signs and symptoms (e.g., constipation, depression, sleep disorders, cognitive dysfunction) [117].

Neuroinflammation is also a feature of PD. Recently, the chemokine fractalkine (CX3CL1), mainly expressed by neurons, has been indicated as a candidate biomarker for PD [118]. CX3CL1 is a modulator of microglial activity that binds to a G-protein-coupled receptor [118] and is responsible for the communication between neurons and glial cells [118]. Along with this, peripheral inflammation, defined by higher circulating levels of IL8, macrophage inflammatory protein (MIP)-1β and by lower concentrations of IL9 and MIP-1α, has been described in older adults with PD [119].

Among the pathogenic mechanisms of neurodegeneration in PD is the accrual of aberrant α-synuclein in dopaminergic neurons, a major component of Lewy bodies, that impairs the activity of mitochondrial complex I [120]. Notably, this inhibitory function, together with α-synuclein overexpression and mutations in genes coding for the mitochondrial regulators Parkin, PINK1, and protein deglycase DJ-1 have been associated with higher ROS production in PD [121,122]. Within this pro-oxidant environment, α-synuclein concurs to promoting oxidative stress, which, in turn, promotes α-synuclein aggregation, thereby installing a vicious circle that ultimately contributes to neurodegeneration [123,124,125,126]. Hence, mitochondrial dysfunction has been deeply implicated in the pathogenesis of familial PD [127]. As a proof of concept, the administration of the synthetic organic compound 6-hydroxydopamine in rodents has shown to be a valuable oxidative stress model for PD as its neurotoxicity mimics many of the hallmarks of PD [128]. 

The involvement of mitochondrial dysfunction in PD was first hypothesized based on the effects of the prodrug 1-methyl-4-phenyl-1,2,3,6-tetrahydropyridine (MPTP) in people with drug addiction [128]. Upon blood-brain barrier crossing and uptake by astrocytes, MPTP is converted into 1-methyl-4-phenylpyridinuim (MPP+), a substrate for dopamine transporter that is selectively uploaded by dopaminergic neurons [128]. Here, MPP+ inhibits the mitochondrial complex I and induces PD-like signs and symptoms [128]. Furthermore, a defective mtDNA homeostasis, including the accumulation of large deletions, has been detected in neuronal cells of the substantia nigra of people with PD [129,130,131]. ROS production in midbrain cells also arises from dopamine metabolism and transport, thus exposing dopaminergic neurons to oxidative damage [132,133,134]. In further support to oxidative stress-driven neuronal loss in PD is the finding of altered levels of glutathione in dopaminergic neurons of transgenic mice, which indicates an impairment in antioxidant defenses in PD [135].

Sporadic forms of PD recapitulate all major hallmarks of aging, thus making PD a prototypical geroscience condition [4,129]. The co-occurrence of mitochondrial dysfunction and neuroinflammation are alleged pathogenic mechanisms of neuronal degeneration [24,136,137]. In particular, defective MQC and DAMPs generation are proposed to be major contributing factors [52]. Indeed, the activation of innate immunity and the consequent inflammation triggered by impaired autophagy and insufficient clearance of damaged mitochondria have been reported in mice depleted of Pink1 or parkin gene (PARK2) [52]. The control of mitophagy over inflammation operates also via the mitophagy mediator Parkin [138]. In particular, Parkin regulates adaptive immunity via mitochondrial antigens presentation to endosomes where they are loaded onto major histocompatibility complex class I molecules [138]. A role similar to mitochondrial antigen presentation is exerted by the intracellular trafficking regulator RAB7A by controlling the fusion of MDVs with late endosomes for subsequent cargo degradation [138]. This additional role of RAB7A ensures mitochondrial antigen presentation in immune cells via MDV trafficking even in the absence of PINK1 or Parkin [138]. Indeed, a decline in PINK1/Parkin expression and activity in PD is implicated in MQC disruption and neuroinflammation via MDV-mediated mitochondrial antigen presentation [138].

Although several lines of evidence indicate a possible link between mitochondrial damage and inflammatory and metabolic disarrangements in PD [137,139], the molecular events the control the sensing of neuronal mitochondrial dysfunction and protein dyshomeostasis at the systemic level are presently unknown. The presence of mitochondrial DAMPs in circulating EVs has been described in older adults with PD and is characterized by a specific inflammatory signature [113]. In particular, higher serum concentrations of small EVs have been found in older people with PD [113]. These EVs included exosomes of endosomal origin that contain mitochondrial signatures [113]. However, after normalizing for the overall serum EV content, people with PD showed lower levels of MDVs relative to non-PD controls [113]. This lower secretion of MDVs in older adults with PD supports the hypothesis of mitochondrial dysfunction secondary to stalling of MQC system [113], especially if considering that the generation of MDV is proposed to be a housekeeping mechanism complementing MQC for preserving cell homeostasis [140].

While placing mitochondrial dysfunction and inflammation among the relevant mechanisms contributing to PD, the analysis of a comprehensive sets of markers and multi-platform statistical approach are needed for capturing PD complexity. Following this strategy, a molecular roadmap including mediators related to MDVs and inflammation has been identified [113]. Among the ever-growing list of molecules linking the two processes, the fibroblast growth factor 21 (FGF21) was associated with PD [113]. Due to its association with impaired MQC in neurons of murine models of tauopathy and prion disease, FGF21 has been indicated as a “mitokine” [141]. Although further analysis in longitudinal cohorts is needed to confirm the discriminatory power of these mediators as PD biomarkers, these findings suggest that scavenging mitochondrial DAMPs, including mtDNA, may offer therapeutic gain in PD.

## 5. Down Syndrome

DS (trisomy 21) is caused by a genetic abnormality and is marked by several features, including cognitive impairment, peculiar craniofacial appearance, gastrointestinal abnormalities, congenital heart defects, and neurological, endocrine and immune disorders [142]. People with DS have shorter lifespan than the general population and DS has mostly been studied as a pediatric condition [142]. However, in light of the increased life expectancy of people with DS, it has become clear that DS is no longer manageable as a pediatric condition [143,144]. When comparing biological vs. chronological age, people with DS age earlier and faster than age-matched controls. Such an accelerated aging is characterized by the early appearance of several phenotypes, including skin wrinkling, graying and loss of hair, visual impairments, early menopause, high prevalence of AD [145], and multimorbidity [146]. These features indicate that DS is a segmental progeroid syndrome [147]. The identification of mediators associated with the occurrence of aging-like phenotypes may therefore represent a powerful tool for the development of effective therapeutic interventions aimed at delaying aging and extending life expectancy.

A set of mediators pertaining to the so-called hallmarks of aging (i.e., DNA methylation, telomere attrition, deregulated nutrient sensing, and immunosenescence) are altered in persons with DS and are invoked as mechanisms involved in premature aging [148,149,150]. When considering the clinical features of DS, attention has been paid to cognitive deficits especially because of their impact on quality of life and prognosis [151,152]. However, musculoskeletal abnormalities have also been described in DS and may contribute to declining physical performance [153,154].

Oxidative stress is a hallmark of segmental progeroid conditions. In DS, this is mainly ascribed to genetic imbalance of chromosome 21, which contains, among others, genes implicated in antioxidant defense, especially SOD1 [11,12]. The supplementation with two natural polyphenols (i.e., epigallocatechin-3-gallate and resveratrol) has shown to counteract oxidative stress in hippocampal progenitor cells from a mouse model of DS via restoring the efficiency of oxidative phosphorylation and stimulating mitochondrial biogenesis [155]. 

Mitochondrial dysfunction has been described in pre-clinical models of DS and in primary cell cultures from people with DS and proposed as a major factor in DS pathogenesis [12,156]. In particular, mitochondrial ROS overproduction has been detected in human skin fibroblasts with trisomic karyotype and has been associated with deficit in mitochondrial complex I, ATP synthase, ADP/ATP translocator, and adenylate kinase activities [157,158]. Mitochondrial DNA mutations and alteration in mtDNA repair systems have also been reported in fibroblasts from persons with DS and DS brain tissue [159,160]. Induced pluripotent stem cells (iPSCs) from people with DS and iPSCs-derived DS neurons show oxidative hallmarks and are more sensitive to oxidative damage than control cells [161,162]. Fragmented and bioenergetically inefficient mitochondria have also been observed in DS as a result of impaired MQC [163]. Along with these changes, signs of premature immunosenescence (i.e., lower activity of natural killer cells, reduced repertoire of T and B lymphocytes, telomere erosion in lymphocytes, and increased risk of developing autoimmune disorders) and a pro-inflammatory profile characterize people with DS [164,165].

However, the pathways involved in the genesis of the pro-oxidant milieu and their intersection with other age-related hallmarks, such as chronic, low-grade inflammation and MQC alterations in DS remain uninvestigated.

## 6. Potential Therapeutics to Counteract Neuroinflammation

The identification of sterile inflammation as a common thread of neurodegenerative diseases has instigated the exploration of pathways involved in innate immunity for the identification of targets amenable for interventions. Indeed, while the release of DAMPs is protective by promoting tissue repair, uncontrolled DAMPs production and consequent hyper-activation of PRRs can lead to neuroinflammation. Therefore, the possibility to target DAMPs and their downstream signaling molecules has been explored as a therapeutic option to attenuate neuroinflammation. 

The neutralization of TLR2 and TLR4 via anti-TLR2 and anti-TLR4 antibodies has shown to block the immune response to fibrillar A*β*(1-42) aggregates in AD models [166]. Also, the abrogation of IL1*β* production by MCC950, a selective NLRP3 inhibitor, or the blockade of IL1 receptor attenuates the propagation of neuroinflammation [167,168], preserves dopaminergic neurons of the substantia nigra, and alleviates motor deficits [169]. 

Immunotherapeutic approaches have also shown potential against PD [170]. These approaches target α-synuclein by reducing or preventing its intracellular accumulation and intercellular transmission [170]. Anti-inflammatory drugs and immunosuppressants limit α-synuclein accrual in PD by inhibiting the release of pro-inflammatory cytokines, restoring lysosome function, and accelerating α-synuclein clearance (reviewed in [170]). Various types of T cells implicated in PD pathogenesis may represent another therapeutic target in PD. In particular, counteracting the development of TH17 cells, known killers of dopaminergic neurons, or blocking the release of IL17 has been proposed as a therapeutic option in PD [171]. 

Resveratrol, a natural polyphenol with anti-inflammatory properties, is currently in clinical trials for AD. This compound mitigates microglia activation by inhibiting TLR4/NF-κB/signal transducer and activator of transcription signaling, and cytokine release upon A*β* stimulation in preclinical models of AD [172]. Epigallocatechin-3-gallate, another well-known natural polyphenol, and resveratrol have also been proposed for the management of DS. In vitro experiments have shown that epigallocatechin-3-gallate is a potent ROS scavenger and an antiapoptotic agent [173]. Among the neuroprotective effects of epigallocatechin-3-gallate are also the activation of mitochondrial biogenesis and the preservation of mitochondrial bioenergetics via Sirtuin 1/AMPK/peroxisome proliferator-activated receptor gamma coactivator 1-alpha (PGC-1α) signaling [155,174,175,176]. The administration of metformin, a drug commonly used for the treatment of type 2 diabetes, has been shown to activate PGC-1α and has been proposed as a potential pharmacological strategy in DS [177]. Indeed, fibroblasts from DS human fetuses treated with metformin showed cristae remodeling and improvement in mitochondrial plasticity and bioenergetics [177]. Finally, melatonin has been proposed as a therapeutic option in DS due to its neuroprotective and anti-neuroinflammatory effects [178]. Melatonin is a modulator of neuronal mitochondrial integrity during aging [178]. However, its efficacy and safety for the management of neuroinflammation warrants investigation.

## 7. Conclusions

Neuroinflammation is a common thread of neurodegenerative disorders. Along with this, mitochondrial dysfunction and derangements in quality control pathways represent major contributors to the accrual of oxidized components within the cells. With the intent of disposing dysfunctional components and unclogging the degradative endo-lysosomal system, cells may unload damaged constituents, including fragmented or whole mitochondria, in the extracellular space. Here, the interaction between bacterial-like motifs of mtDNA and cells of innate immunity may trigger inflammation. Thus, an enhancement of EV secretion can be a protective strategy as it may help alleviate the disruption of the endosomal–lysosomal system in disease-vulnerable neurons. The characterization of the cargo of these EVs may assist in clarifying the pathways involved in neuronal degeneration and support the development of therapeutic approaches sustaining neuronal exosome biogenesis in the setting of several neurodegenerative disorders.
